# Olezarsen: A Next-Generation Antisense Therapy for Hypertriglyceridemia and Familial Chylomicronemia Syndrome

**DOI:** 10.7759/cureus.96715

**Published:** 2025-11-12

**Authors:** Jace Joshy, Anna Javed, Manish Kumar Singh, Bhanu Priya Singh, Ritesh Kumar, Sartaj Hussain

**Affiliations:** 1 Pharmacology, All India Institute of Medical Sciences, Vijaypur, Vijaypur, IND; 2 Biochemistry, Government Medical College Badaun, Sirsauli, IND; 3 Pharmacology, Shri Mata Vaishno Devi Institute of Medical Excellence, Katra, IND

**Keywords:** antisense oligonucleotides, familial chylomicronemia syndrome, hypertriglyceridemia, olezarsen, tryngolza

## Abstract

Olezarsen (Tryngolza) is a next-generation, N-acetylgalactosamine (GalNAc)-conjugated antisense oligonucleotide that selectively inhibits hepatic ApoC-III, a key regulator of triglyceride metabolism. By inhibiting ApoC-III, olezarsen increases triglyceride clearance through both lipoprotein lipase (LPL)-dependent and -independent pathways. In the Phase 3 BALANCE trial, olezarsen reduced fasting triglycerides by approximately 60% at 12 months in patients with familial chylomicronemia syndrome (FCS), with a marked decrease in pancreatitis events versus placebo. Consistent triglyceride reductions (around 50%) were also observed in moderate and severe hypertriglyceridemia, along with improvements in ApoB-containing lipoproteins and high-density lipoprotein (HDL) profiles.

In completed trials, olezarsen demonstrated a favorable safety profile, with most adverse events limited to mild injection-site reactions and no clinically significant thrombocytopenia. Ongoing Phase 3 trials (ESSENCE, CORE, and CORE2) will further define its role in cardiovascular risk reduction and pancreatitis prevention in broader hypertriglyceridemic populations. Olezarsen represents a precision medicine advance, offering effective triglyceride lowering with improved tolerability compared with earlier antisense therapies.

## Introduction and background

Hypertriglyceridemia is a metabolic condition characterized by elevated levels of triglycerides in the bloodstream, defined as fasting triglycerides greater than 150 mg/dL (>1.7 mmol/L) [1]. Chronic hypertriglyceridemia is an important risk factor for life-threatening complications such as atherosclerotic cardiovascular disease (ASCVD) and acute pancreatitis, particularly when triglyceride levels exceed 500 mg/dL (≥5.6 mmol/L) [2]. Globally, it is estimated that about 27% of adults have elevated triglyceride levels (>150 mg/dL), while roughly 1-2% have severe hypertriglyceridemia (≥500 mg/dL), which notably increases the risk of pancreatitis and other related complications [3].

Current treatment options for hypertriglyceridemia include statins (HMG-CoA reductase inhibitors), which reduce triglycerides by approximately 10-30%, primarily by decreasing hepatic LDL production [2,4]; fibrates (PPAR-α agonists), which lower triglycerides by about 20-30% through activation of lipoprotein lipase (LPL) [5,6]; niacin (nicotinic acid), which reduces triglycerides by 20-50% by reducing free fatty acid release from adipose tissue [7]; omega-3 fatty acids (eicosapentaenoic acid (EPA) ± docosahexaenoic acid (DHA)), which can reduce triglycerides by more than 30% by reducing hepatic triglyceride synthesis and very-low-density lipoprotein (VLDL) production [8]; and Saroglitazar, a dual PPAR-α/γ agonist, lowers triglycerides by approximately 30-50%, improves non-HDL-C and VLDL-C levels, and offers modest glycemic benefits through PPAR-γ-mediated effects [9].

Familial chylomicronemia syndrome (FCS) is a rare autosomal recessive condition characterized by defective or absent LPL activity, most commonly caused by mutations in genes such as LPL, APOC2, APOA5, GPIHBP1, or LMF [10]. This defect leads to persistent, severe hypertriglyceridemia (>1,000 mg/dL) and a increased risk of recurrent, life-threatening pancreatitis [11]. The cornerstone of FCS management is a strict low-fat diet; however, dietary interventions alone rarely reduce triglycerides to below 500 mg/dL, the threshold necessary to prevent acute pancreatitis [12,13]. Standard triglyceride-lowering drugs, including statins, fibrates, niacin, and omega-3 fatty acids, are generally ineffective in FCS due to the underlying LPL dysfunction [14].

Volanesorsen (Waylivra), an earlier ApoC-III-targeted antisense oligonucleotide, received conditional approval in the European Union in May 2019 for adults with genetically confirmed FCS who were at high risk of pancreatitis [15]. However, it was not approved by the US FDA due to safety concerns, primarily thrombocytopenia [16].

Olezarsen (Tryngolza) is a next-generation antisense oligonucleotide developed by Ionis Pharmaceuticals, and it received FDA approval in December 2024 for the treatment of adults with FCS [17,18]. It offers a more targeted, efficacious, and safer therapeutic approach compared with conventional therapies. This review provides an overview of olezarsen’s discovery and development, mechanism of action, clinical efficacy, safety profile, and potential broader clinical applications.

From a translational standpoint, genetic evidence strongly supports ApoC-III as a therapeutic target. Rare loss-of-function mutations in the APOC3 gene confer lifelong low triglyceride levels (approximately 40-50% lower than average) and are associated with approximately 40% reduction in coronary heart disease risk [19]. The development of olezarsen was directly informed by these insights, effectively translating the protective effect of APOC3 deficiency into a therapeutic strategy for the treatment of hypertriglyceridemia.

The aim of this narrative review is to synthesize current translational, pharmacologic, and clinical trial evidence to determine whether olezarsen, a GalNAc-conjugated ApoC-III antisense oligonucleotide, can safely extend the proven benefits of ApoC-III inhibition from FCS to broader hypertriglyceridemic populations.

## Review

Data sources

The search methodology followed a narrative, evidence-integrated review design focused on evaluating olezarsen, an antisense inhibitor of ApoC-III, in the management of FCS and severe hypertriglyceridemia. Literature was identified through PubMed/MEDLINE, Embase, and the Cochrane Library, supplemented by data from ClinicalTrials.gov, the EU Clinical Trials Register, FDA and other regulatory submissions, manufacturer communications, and conference abstracts. The search covered publications and records from January 1, 2000 to August 31, 2025. Search terms included “olezarsen,” “IONIS-APOCIII-LRx,” “APOC3-LRx,” “Tryngolza,” “apolipoprotein C-III,” “APOC3,” “familial chylomicronemia syndrome,” “severe hypertriglyceridemia,” “volanesorsen,” and “plozasiran” or “ARO-APOC3,” combined using Boolean operators (AND/OR).

Studies were included if they met the following criteria: adult participants (≥18 years) with FCS or fasting triglyceride levels ≥150 mg/dL; interventions involving olezarsen at any dose or schedule; study designs encompassing Phase 1-3 clinical trials, open-label extensions, or regulatory/industry reports containing human efficacy or safety data; outcomes measuring changes in triglycerides and/or ApoC-III, incidence of pancreatitis, or safety parameters; and publications available in English. Exclusion criteria included preclinical or animal-only studies, commentaries without primary data, trials focusing on other RNA-based lipid agents, and non-English publications lacking regulatory documentation.

Structure

Olezarsen is a second-generation antisense oligonucleotide (ASO) designed to target hepatic APOC3 mRNA, thereby inhibiting ApoC-III protein production [20]. This ASO consists of a 20-nucleotide, single-stranded DNA-like molecule with a phosphorothioate backbone that confers resistance to nuclease degradation [21]. The sequence is complementary to APOC3 mRNA, enabling specific binding via Watson-Crick base pairing. Second-generation ASOs incorporate 2'-O-methoxyethyl (MOE) modifications on the ribose sugars to enhance binding affinity and specificity and to prolong the oligonucleotide half-life [22]. Olezarsen is conjugated to a triantennary N-acetylgalactosamine (GalNAc3) ligand, which facilitates liver-specific uptake by binding to asialoglycoprotein receptors on hepatocytes, dramatically improving delivery efficiency [23]. Olezarsen is formulated as a sodium salt for increased solubility; its molecular formula is C₂₉₆H₄₁₉N₇₁O₁₅₄P₂₀S₁₉Na₂₀, and its molecular weight is approximately 9,124.5 Daltons [17].

Mechanism of action

Olezarsen is a GalNAc3-conjugated ASO that targets ApoC-III mRNA in hepatocytes, thereby reducing ApoC-III protein production [23-25]. ASOs are short, synthetic, single-stranded nucleic acids that bind to complementary mRNA sequences and promote their degradation [26]. Upon binding to APOC3 mRNA, olezarsen triggers RNase H1-mediated mRNA cleavage, preventing translation of the ApoC-III protein [27]. ApoC-III is a key protein that normally raises triglyceride levels by doing two things: it reduces the activity of LPL and slows down the liver’s ability to remove triglyceride-rich lipoproteins (TRLs) from the blood [28]. When olezarsen lowers ApoC-III levels, this “brake” on fat breakdown is released. As a result, TRLs are cleared more efficiently, not only through the usual LPL-dependent pathway but also through alternative LPL-independent routes, such as improved receptor-mediated uptake by the liver. This means that even in patients with a defective LPL enzyme, triglycerides can still be metabolized [24,29,30]. This targeted mechanism addresses the fundamental defect in FCS, impaired chylomicron clearance, and underlies olezarsen’s high efficacy in this syndrome.

Pharmacokinetics

Olezarsen is administered as an 80 mg subcutaneous injection once monthly [11]. The drug reaches its maximum plasma concentration (Tₘₐₓ) in approximately 2 hours, indicating rapid uptake from the injection site into the bloodstream [17].

Due to its GalNAc3-mediated hepatic targeting, olezarsen exhibits extensive tissue uptake, particularly in the liver and kidneys, and a large apparent volume of distribution [23,25]. The apparent central volume is approximately 92 L, and the peripheral volume is around 2960 L, with >99% plasma protein binding [31]. At the 80 mg dose, the mean steady-state peak concentration (C_max_) is approximately 883 ng/mL, and the area under the curve (AUC_τ_) is about 7440 ng·h/mL. Exposure (C_max_ and AUC) increases approximately proportionally over the 10-120 mg dose range [17].

Olezarsen is metabolized in hepatocytes by endo- and exonucleases, which cleave the antisense oligonucleotide into shorter fragments for elimination [17,31].

It has a terminal elimination half-life of around 4 weeks, consistent with once-monthly dosing. Clearance is primarily metabolic, with <1% of the drug excreted unchanged in urine per 24 hours, and no significant accumulation observed with repeated dosing [17,31].

Key pharmacokinetic parameters (80 mg dose) of olezarsen are presented in Table 1 [17].

**Table 1:** Key pharmacokinetic parameters of olezarsen following an 80 mg dose.

Parameter	Value	Notes/Description
Dose	80 mg	Single dose
Peak plasma concentration (C_max_)	~883 ng/mL	Maximum observed plasma concentration
Time to peak (T_max_)	~2 hours	Time taken to reach C_max_
Exposure (AUC_τ_)	~7440 ng·h/mL	Area under the plasma concentration–time curve per dosing interval
Apparent central volume (V_c_)	~92 L	Central compartment volume of distribution
Peripheral volume (V_p_)	~2960 L	Peripheral compartment volume of distribution
Plasma protein binding	>99%	Highly bound to plasma proteins
Terminal half-life	~4 weeks	Indicates prolonged systemic exposure
Renal excretion	<1% unchanged in urine	Minimal renal elimination within 24 hours

Pharmacodynamics

In patients with FCS, once-monthly 80 mg olezarsen produces a rapid and profound reduction in ApoC-III levels: approximately 57% at 1 month, 69% at 3 months, 72% at 6 months, and 80% at 12 months [18]. These reductions in ApoC-III translate into marked decreases in triglyceride levels and a lower incidence of pancreatitis [17].

Beyond triglyceride lowering, ApoC-III inhibition appears to remodel lipoprotein profiles in a beneficial way. Nuclear magnetic resonance (NMR) spectroscopy showed that olezarsen therapy (50 mg monthly) reduced total TRL particles by ~51% and favorably remodeled LDL subclasses, increasing large LDL by ~186% while reducing small, dense LDL by ~39% [32]. HDL particle concentration also increased (~15% overall, driven by a rise in small HDL particles), reflecting an overall improvement in the atherogenic lipid profile [32]. These changes suggest that olezarsen not only lowers plasma triglyceride levels but may also favorably alter lipoprotein composition, an effect that could enhance its cardiovascular (CV) benefit.

Clinical efficacy and therapeutic indications

Familial Chylomicronemia Syndrome (FCS)

Olezarsen (Tryngolza) was the first medication approved (in 2024) specifically for adult patients with FCS [18]. It is administered once monthly by subcutaneous injection, in conjunction with a strict low-fat diet. In the Phase 3 BALANCE trial (NCT04568434), the 80 mg dose of olezarsen reduced fasting triglyceride levels by approximately 44% at 6 months and 59% at 12 months compared with placebo, while ApoC-III levels dropped by over 70%, confirming the mechanism of action [11]. Notably, by 12 months, only one acute pancreatitis event occurred in each of the olezarsen arms (80 mg and 50 mg) versus 11 events in the placebo group, highlighting a dramatic reduction in this life-threatening complication [11]. Although long-term outcomes beyond one year are not yet available, olezarsen represents a significant advancement in the management of FCS.

Moderate Hypertriglyceridemia and CV Risk Reduction

In patients with moderate hypertriglyceridemia (150-499 mg/dL) at high CV risk, olezarsen has shown potential to improve lipid profiles. The Phase 2b BRIDGE-TIMI 73a trial (NCT05355402) demonstrated that monthly doses of 50 mg or 80 mg olezarsen reduced triglycerides by ~50% and also lowered ApoC-III, apolipoprotein B (ApoB), and non-HDL cholesterol by ~20%, without increasing LDL cholesterol [33]. The ongoing Phase 3 ESSENCE-TIMI 73b trial (NCT05610280) has enrolled over 1,400 adults with ASCVD or high CV risk, comparing olezarsen 50 mg and 80 mg versus placebo over 12 months and including a coronary CT angiography sub-study [34]. In May 2025, Ionis announced that ESSENCE met its primary endpoint: olezarsen achieved a 58-61% triglyceride reduction at 6 months (for the 50 mg and 80 mg doses, respectively) versus placebo, and all key secondary endpoints were met with a favorable safety profile [35]. Full results of ESSENCE, including effects on coronary plaque, are expected in late 2025. If these improvements in triglycerides and ApoB-containing lipoproteins translate into plaque stabilization or regression, it would reinforce the case for ApoC-III inhibition as a strategy to reduce CV events. The ESSENCE trial’s outcomes will help determine whether olezarsen can slow plaque progression and lower the risk of future CV events.

Hypertriglyceridemia and Pancreatitis Prevention

Severe hypertriglyceridemia (TG ≥500 mg/dL) is relatively common in individuals with metabolic syndrome (such as those with diabetes or obesity) or certain inherited disorders. At extremely high levels (>1,000 mg/dL), the risk of acute pancreatitis rises substantially. Olezarsen is being evaluated in this broader population to prevent pancreatitis by aggressively lowering triglyceride levels. Two parallel Phase 3 trials are underway: CORE-TIMI 72a (NCT05079919), which is testing olezarsen 80 mg versus placebo, and CORE2-TIMI 72b (NCT05552326), which is testing olezarsen 50 mg versus placebo, each in patients with severe hypertriglyceridemia (≥500 mg/dL) [36]. Together, these trials include approximately 1,000 participants. The primary endpoint is the percent change in triglycerides at 6 months, with secondary endpoints including 12-month triglyceride reduction, pancreatitis incidence, and hepatic fat changes. Results from CORE and CORE2 are anticipated in late 2025 or early 2026. An open-label extension study (CORE-OLE, NCT05681351) is also recruiting to evaluate up to three years of long-term safety and efficacy in participants from the CORE trials [37]. It is noteworthy that no prior triglyceride-lowering therapy has demonstrated a reduction in pancreatitis incidence in a controlled trial [11]. If the CORE studies show that olezarsen’s robust triglyceride reduction leads to significantly fewer pancreatitis episodes in the general severe hypertriglyceridemia population, it would represent a groundbreaking clinical advance in preventive care for pancreatitis.

Clinical studies

An updated overview of the relevant clinical trials (as of August 2025) is provided in Table 2.

**Table 2:** Overview of olezarsen (ApoC-III antisense inhibitor) trials: design, endpoints, outcomes, and key limitations. ApoC-III: Apolipoprotein C-III; APOC3: Gene encoding apolipoprotein C-III; apoB: Apolipoprotein B; ASCVD: Atherosclerotic cardiovascular disease; AST: Aspartate aminotransferase; ALT: Alanine aminotransferase; AUC_τ_: Area under the plasma concentration-time curve per dosing interval; CTA: Coronary computed tomography angiography; CV: Cardiovascular; CVD: Cardiovascular disease; C_max_: Maximum observed plasma concentration; FCS: Familial chylomicronemia syndrome; HDL-C: High-density lipoprotein cholesterol; HTG: Hypertriglyceridemia; LDL-C: Low-density lipoprotein cholesterol; Lp(a): Lipoprotein(a); mo: Months; N: Sample size; NCT: National clinical trial number; non-HDL-C: Non-high-density lipoprotein cholesterol; PD: Pharmacodynamics; PK: Pharmacokinetics; QW: Once weekly; Q2W: Once every two weeks; Q4W: Once every four weeks; SC: Subcutaneous; TC: Total cholesterol; TG: Triglycerides; T_max_: Time to reach maximum plasma concentration; V_c_: Apparent central volume of distribution; V_p_: Apparent peripheral volume of distribution; VLDL-C: Very-low-density lipoprotein cholesterol.

Trial name	Study population	Sample size & design	Endpoints	Results	Quality appraisal / key limitations
Safety, tolerability, PK, and PD of IONIS-APOCIII-LRx (Phase 1) NCT02900027 [38]	Healthy volunteers with TG levels ≥90 mg/dL or ≥200 mg/dL	56 adults. Single-dose cohorts: received 10, 30, 60, 90, or 120 mg SC injection. Multiple-dose cohorts: received 15 or 30 mg weekly for 6 weeks, or 60 mg every 4 weeks for 3 months.	Primary: safety and tolerability of olezarsen. Secondary: pharmacokinetics and pharmacodynamic effects of olezarsen.	Completed. Well tolerated across single- and multiple-dose cohorts. No platelet count reductions; cumulative doses did not show AST/ALT increases. Route: subcutaneous injection (80 mg once monthly). T_max_: ~2 hours (rapid absorption). C_max_: ~883 ng/mL (steady state). AUC_τ_: ~7440 ng·h/mL. Distribution: large (V_c _~92 L; V_p_ ~2960 L); >99% protein bound. Half-life: ~4 weeks. Metabolism: hepatic endo/exonucleases. Excretion: <1% unchanged in urine.	Very small, short follow-up; surrogate outcomes only; healthy/mild HTG → limited external validity to FCS/severe HTG; sponsor-run study.
Study of ISIS 678354 (AKCEA-APOCIII-LRx) in participants with hypertriglyceridemia and established cardiovascular disease (CVD) (Phase 2) NCT03385239 [39]	Fasting TG 200-500 mg/dL and established CVD or high cardiovascular risk	114 adults. Dosing cohorts: 10 mg Q4W, 50 mg Q4W, 15 mg Q2W, 10 mg QW, and placebo.	Primary: % change in fasting TG from baseline to 6 months. Secondary: % change in ApoC-III, VLDL-C, non-HDL-C, HDL-C, LDL-C, and TC.	Completed. At 6 months: TG ↓ 23% (10 mg Q4W), 56% (15 mg Q2W), 60% (10 mg QW), 60% (50 mg Q4W) vs +6% (placebo) (p<0.001). ApoC-III ↓ up to 74%, HDL-C ↑ 29-40%, non-HDL-C ↓ up to 24%, VLDL-C ↓ ~60%. Well tolerated.	6-month duration; powered for TG, not clinical events; mixed background therapies.
BRIDGE-TIMI 73a (Phase 2b) NCT05355402 [33]	Moderate HTG (150-499 mg/dL) + high CV risk; severe HTG (≥500 mg/dL)	154 adults. 1:1 randomization to 50 or 80 mg; then 3:1 olezarsen vs placebo Q4W for 6 months.	Primary: % change in TG at 6 months. Secondary: % achieving TG <500 mg/dL, ApoC-III, apoB.	Completed. At 6 months: TG ↓ 49.3% (50 mg) and 53.1% (80 mg) vs placebo (p<0.0001). 89% achieved TG <500 vs 28% (placebo). ApoC-III ↓ ~70%, apoB ↓ 20%. Well tolerated.	Short duration (6 months); surrogate endpoints; enriched/high-care cohort → limited generalizability; small N per stratum → less precise safety estimates.
BALANCE (Phase 3) NCT04568434 [11]	FCS; fasting TG ≥880 mg/dL (mean ~2600 mg/dL)	66 adults randomized 1:1:1 to olezarsen 80 mg, 50 mg Q4W, or placebo during Weeks 1-49 of the 53-week treatment period.	Primary: % change in fasting TG at 6 months. Secondary: pancreatitis incidence, ApoC-III, platelets, long-term TG (12 months).	Completed. At 6 months: TG ↓ 43.5% (80 mg; p=0.0009) and 22.4% (50 mg; p=0.0775). At 12 months: TG ↓ 59.4% (80 mg; p=0.0002) and 43.8% (50 mg; p=0.0044). ApoC-III ↓ 73.7% (80 mg) and 65.5% (50 mg). Pancreatitis: 1 (80 mg), 1 (50 mg) vs 11 (placebo). No platelet drop <50k. Favorable tolerability.	Ultra-rare disease → small sample (~66); pancreatitis analysis based on few events → wide CI; sponsor-assayed outcomes; 1-year follow-up only.
CORE-TIMI 72a (Phase 3) NCT05079919 [36]	Severe HTG (TG ≥500 mg/dL), non-FCS population	617 adults. 2:1 randomization to olezarsen 80 mg Q4W vs placebo for 12 months.	Primary: % change in TG at 6 months. Secondary: long-term TG (12 months), hepatic fat fraction, pancreatitis.	Ongoing. Results expected in late 2025 or early 2026.	Heterogeneous secondary HTG → variable TG response; powered for TG only; potential attrition risk.
CORE2-TIMI 72b (Phase 3) NCT05552326 [36]	Severe HTG (TG ≥500 mg/dL)	446 adults. 2:1 randomization to olezarsen 50 mg Q4W vs placebo for 12 months.	Primary: % change in TG at 6 months. Secondary: long-term TG (12 months), hepatic fat fraction, pancreatitis.	Ongoing. Results expected in late 2025 or early 2026.	Same limitations as CORE-TIMI 72a; lower dose may show smaller effect; pancreatitis secondary → underpowered.
ESSENCE-TIMI 73b (Phase 3) NCT05610280 [34]	Moderate HTG (200-500 mg/dL) with ASCVD or high CV risk (~9% TG ≥500 mg/dL)	1,478 adults randomized to olezarsen 50 mg, 80 mg Q4W, or placebo; 6-month primary and 12-month total duration; CTA sub-study.	Primary: % change in TG at 6 months. Secondary: change in non-calcified coronary plaque (CTA).	Top-line results announced. Olezarsen met all primary and secondary endpoints. At 6 months: TG ↓ 58% (50 mg) and 61% (80 mg) vs placebo (p<0.0001). CTA imaging data not yet published.	Primary endpoint biochemical, not clinical; CTA sub-study prone to selection/reader bias; background statins may inflate incremental benefit; CV/pancreatitis outcomes require longer follow-up.
CORE-OLE (Open-Label Extension) NCT05681351 [37]	Severe HTG participants from CORE and CORE2 trials	Participants from parent trials rolled over to open-label treatment with olezarsen (ongoing).	Primary: % change in lab values from baseline to Week 157; % of adverse events.	Ongoing. Currently recruiting.	Uncontrolled, open-label design.

Safety and tolerability

Olezarsen has demonstrated an overall favorable safety profile in clinical trials, with adverse events that were mostly mild to moderate and not significantly higher in incidence than placebo. Notably, unlike its predecessor volanesorsen, olezarsen has not been associated with severe thrombocytopenia. The most common side effects included injection-site reactions (≈19%; redness, itching, or pain), mild platelet count reductions (≈12%; no cases of platelets <50,000/μL), and joint pain or arthralgia (≈9%).

Rare hypersensitivity reactions such as flushing, chills, chest tightness, or shortness of breath have been reported, though these events were infrequent. Furthermore, no serious issues such as severe thrombocytopenia, bleeding, or organ toxicity have been observed with olezarsen. Mild, transient elevations in liver enzymes have occurred, while renal function has remained stable. There have been no reports of lipoatrophy (localized fat loss at injection sites) or other injection-site tissue damage. In fact, inflammatory markers often improved with ApoC-III reduction [11].

There are no clinical data on olezarsen use in pregnancy, breastfeeding, or the pediatric population; therefore, its use is not recommended in these groups [17]. Olezarsen does not significantly inhibit or induce cytochrome P450 enzymes and does not displace highly protein-bound drugs (e.g., warfarin or ibuprofen) from plasma proteins. Even at supratherapeutic doses (1.5× the maximum recommended), it did not cause any clinically relevant QT interval prolongation [17]. Given its stable safety profile, intensive monitoring (as required for volanesorsen) is generally unnecessary, although baseline and periodic checks of platelet counts and liver enzymes are still advised.

In contrast to volanesorsen, which was associated with significant thrombocytopenia and required frequent platelet monitoring, olezarsen’s hepatocyte-targeted design has reduced hematologic side effects [14,39]. This is likely because GalNAc-mediated hepatic uptake allows equivalent ApoC-III knockdown at much lower systemic antisense oligonucleotide exposure, minimizing off-target interactions with platelets and the reticuloendothelial system. Consequently, clinically meaningful platelet declines have been infrequent and reversible in completed studies, supporting a less intensive monitoring schedule than that recommended for first-generation ApoC-III antisense oligonucleotides [39]. As a result, patients treated with olezarsen do not require the same level of laboratory surveillance, greatly simplifying outpatient management. Overall, olezarsen is well tolerated and can be safely managed in outpatient settings.

Access and cost

Olezarsen (Tryngolza) became available in the U.S. in early 2025 with a wholesale acquisition cost of approximately $595,000 per year [40,41]. Distribution is restricted to a single specialty pharmacy outlet (PANTHERx Rare). The manufacturer offers copayment assistance programs for commercially insured patients, reducing out-of-pocket costs [42]. However, patients with government insurance are not eligible for such programs, creating significant access barriers.

Limitations

The current evidence on olezarsen has several limitations. Many trials had relatively short follow-up durations (≤12 months), so long-term efficacy and safety are not yet fully established. Additionally, most of the data come from industry-sponsored studies, and some results have only been released through press announcements, introducing potential reporting bias. Furthermore, the high cost and restricted distribution of olezarsen impose practical barriers that may limit its real-world impact despite its proven efficacy. While olezarsen has demonstrated impressive reductions in triglyceride levels, it remains uncertain whether these changes will translate into meaningful reductions in heart attacks, strokes, or mortality. To date, no CV outcomes trial has been completed for this drug, and current evidence is limited to improvements in surrogate markers such as plaque burden or lipid profiles. These encouraging signals need confirmation from studies that assess actual clinical outcomes. In addition, careful long-term follow-up will be important to rule out delayed safety concerns, for example, the potential for increased liver fat due to the sustained diversion of triglyceride-rich particles into the liver. Thus far, however, clinical trials have not observed significant hepatic steatosis.

Translational implications

Olezarsen represents a precision medicine approach by selectively inhibiting ApoC-III, a key regulator identified through human genetics, rather than broadly altering lipid metabolism. Its strong triglyceride-lowering effect and clinical benefit in FCS validate APOC3 as a therapeutic target. As a GalNAc-conjugated antisense oligonucleotide, olezarsen also demonstrates how targeted delivery improves both efficacy and safety compared with earlier agents such as volanesorsen [43]. More broadly, ApoC-III inhibition offers a strategy to address residual cardiometabolic risk, including persistent hypertriglyceridemia despite statin therapy.

Olezarsen belongs to a new wave of RNA-based therapies being developed to treat disorders of lipid metabolism. Its progress mirrors that of other antisense and RNA interference (RNAi) drugs aimed at different targets, including lipoprotein(a) and ANGPTL3. For example, pelacarsen, an antisense therapy directed against lipoprotein(a), is already in a Phase 3 trial to test whether lowering Lp(a) can prevent heart attacks and strokes [44]. Similarly, another ApoC-III-directed therapy, the siRNA agent plozasiran (ARO-APOC3), has shown promising triglyceride-lowering effects in early studies [45]. Together, these efforts represent a major shift in CV medicine: translating genetic and molecular insights into precisely targeted therapies. If ongoing trials demonstrate that lowering these specific lipid particles truly reduces clinical events, it could mark the beginning of a new era in preventive cardiometabolic care.

Future research directions

Future studies must clarify whether olezarsen’s triglyceride-lowering effects translate into fewer CV events or reduced mortality. While early data suggest benefits in reducing pancreatitis, only long-term outcome trials and post-marketing registries can confirm its impact on major clinical events. Real-world evidence will also be key in defining safety, durability, and healthcare outcomes. Important questions remain, such as whether lower or less frequent dosing can sustain efficacy, how combination therapy might work in refractory patients, and which subgroups derive the greatest clinical benefit. In FCS, early intervention may help prevent pancreatitis altogether, but pediatric use requires dedicated evaluation. Priority areas include: (i) a pediatric pharmacokinetic/pharmacodynamic and safety program in genetically confirmed FCS; (ii) a CV outcomes trial in high-risk hypertriglyceridemia comparing olezarsen with the best standard lipid-lowering therapy; and (iii) dose-de-escalation or extended-interval studies (e.g., every 8-12 weeks) to define the minimal effective maintenance schedule. Additional studies should evaluate olezarsen in rational combinations (e.g., high-dose omega-3 fatty acids, fenofibrate, or emerging ANGPTL3 inhibitors) in patients who fail to achieve triglyceride levels <500 mg/dL, and should include systematic hepatic fat and glucose-insulin phenotyping to rule out long-term metabolic liabilities.

Beyond clinical outcomes, olezarsen’s development signals broader advances in RNA therapeutics. Its improved tolerability compared with volanesorsen highlights important lessons for safer oligonucleotide design, while next-generation chemistry may allow longer dosing intervals or alternative delivery routes. Research may also reveal benefits beyond lipid metabolism, including potential effects on insulin sensitivity or fat distribution. At the same time, cost and access will shape clinical adoption, health economic studies and real-world data on reduced hospitalizations may help justify payer coverage.

Together, these lines of research will define olezarsen’s role and guide the next generation of targeted cardiometabolic therapies.

## Conclusions

Olezarsen is a significant advance in the treatment of severe hypertriglyceridemia, particularly in patients with FCS. By targeting ApoC-III, it effectively lowers triglyceride levels and thereby reduces the risk of acute pancreatitis. Olezarsen also shows promise for patients whose hypertriglyceridemia is not controlled by lifestyle modification or existing therapies, improving lipid parameters associated with cardiovascular risk. Ongoing trials, such as CORE and ESSENCE, will further clarify its impact on cardiovascular outcomes. Its RNA-targeted mechanism, combined with convenient once-monthly dosing and a generally mild side-effect profile, makes it suitable for long-term therapy. Alongside dietary management and other lipid-lowering interventions, olezarsen offers a novel and effective option for FCS and severe hypertriglyceridemia. However, patient selection, high treatment cost, and insurance coverage considerations will likely influence its real-world uptake. Overall, olezarsen exemplifies the progress of precision medicine in addressing the root molecular causes of complex metabolic disorders.
